# Lupus Profundus: A Case Report from Pakistan

**DOI:** 10.7759/cureus.2697

**Published:** 2018-05-28

**Authors:** Amber Siddiqui, Haseeb A Bhatti, Javeria Ashfaq

**Affiliations:** 1 Dow University of Health Sciences, Jinnah Sindh Medical University (SMC); 2 Department of Internal Medicine, Jinnah Postgraduate Medical Centre, Karachi, PAK; 3 Medicine Ward 3, Jinnah Postgraduate Medical Centre, Karachi, PAK

**Keywords:** lupus profundus, cutaneous lupus, systemic lupus erythematosus (sle), autoimmunity, pakistan, cutaneous ulcerative lesions, lupus erythematosus

## Abstract

Lupus erythematosus (LE) is termed as an autoimmune chronic condition which involves a spectrum of symptoms. It is a part of the connective tissue diseases. Its cutaneous form is termed as cutaneous lupus erythematosus (CLE). Prevalence of CLE is about 70 cases per 100,000 persons. The least common variety of CLE is lupus profundus (LP)—only 5% of cases. Lupus profundus, although rare, must be kept in the differential diagnoses of ulcerated lesions. It may present as a localized entity or in association with systemic lupus erythematosus (SLE) or it may lead to SLE later in life. Early diagnosis based on histopathology and aggressive treatment is essential to prevent significant physical morbidity and progression to systemic involvement. We report a case of biopsy-proven lupus profundus in a 40-year-old female who presented with high-grade fever and multiple ulcerated lesions. The lesions were appreciated on the left thigh, right gluteus, and left arm. They had an erythematous base and edematous necrotizing centers with purulent discharge. She had a history of oral ulcers, joint pain, photosensitivity, dyspnea, peptic ulcer disease, and signs of depression. Her autoimmune assays were unremarkable. We treated her with antibiotics, oral hydroxychloroquine, and oral corticosteroid. Potassium permanganate wash and methylprednisolone aceponate were applied locally on the lesions. Ulcerated LP is a rare cause of ulcerated/indurated, painful subcutaneous plaques. It may present as a localized entity or in association with SLE or it may lead to SLE later in life.

## Introduction

Lupus erythematosus (LE) is termed as an autoimmune and chronic condition which involves a spectrum of symptoms. It is a part of the connective tissue diseases. The systemic form of LE is called systemic lupus erythematosus (SLE) whereas the cutaneous form is termed as cutaneous lupus erythematosus (CLE). SLE and CLE can coexist as well as exist as separate entities. CLE can also be the precursor of SLE in some cases [1].

Prevalence of CLE is about 70 cases per 100,000 persons in United States. The least common variety of CLE is lupus profundus (LP)—only 5% cases, followed by subacute CLE (SCLE)—15% cases, and the most common is discoid lupus erythematosus (DLE)—80% cases [1]. While LP mostly involves the plantar surfaces, occurence of ulcerated LP lesions on non-acral sites is uncommon [2].

## Case presentation

 We report a case of a 40-year-old female admitted in October 2017, in a tertiary care hospital in Karachi, Pakistan, with skin lesions for three months and fever for one week.

The first lesion appeared on her left thigh, 3 months back, 3x4 cm in size; it was itchy, tender, red, and swollen. After initial 1-2 days, there was purulent discharge from the lesion, followed by watery discharge, and slight bleeding. It then faded with scarring and induration within a few weeks. One week previously, she developed a fever which was documented to be 102-103°F, intermittent, relieved by antipyretics, and associated with rigors and chills. There was a history of oral ulcers, joint pain, photosensitivity, dyspnea, peptic ulcer disease, and signs of depression. However, none of these signs could be appreciated at the time of presentation. Family history was positive for the consanguineous marriage of the parents.

**Figure 1:**
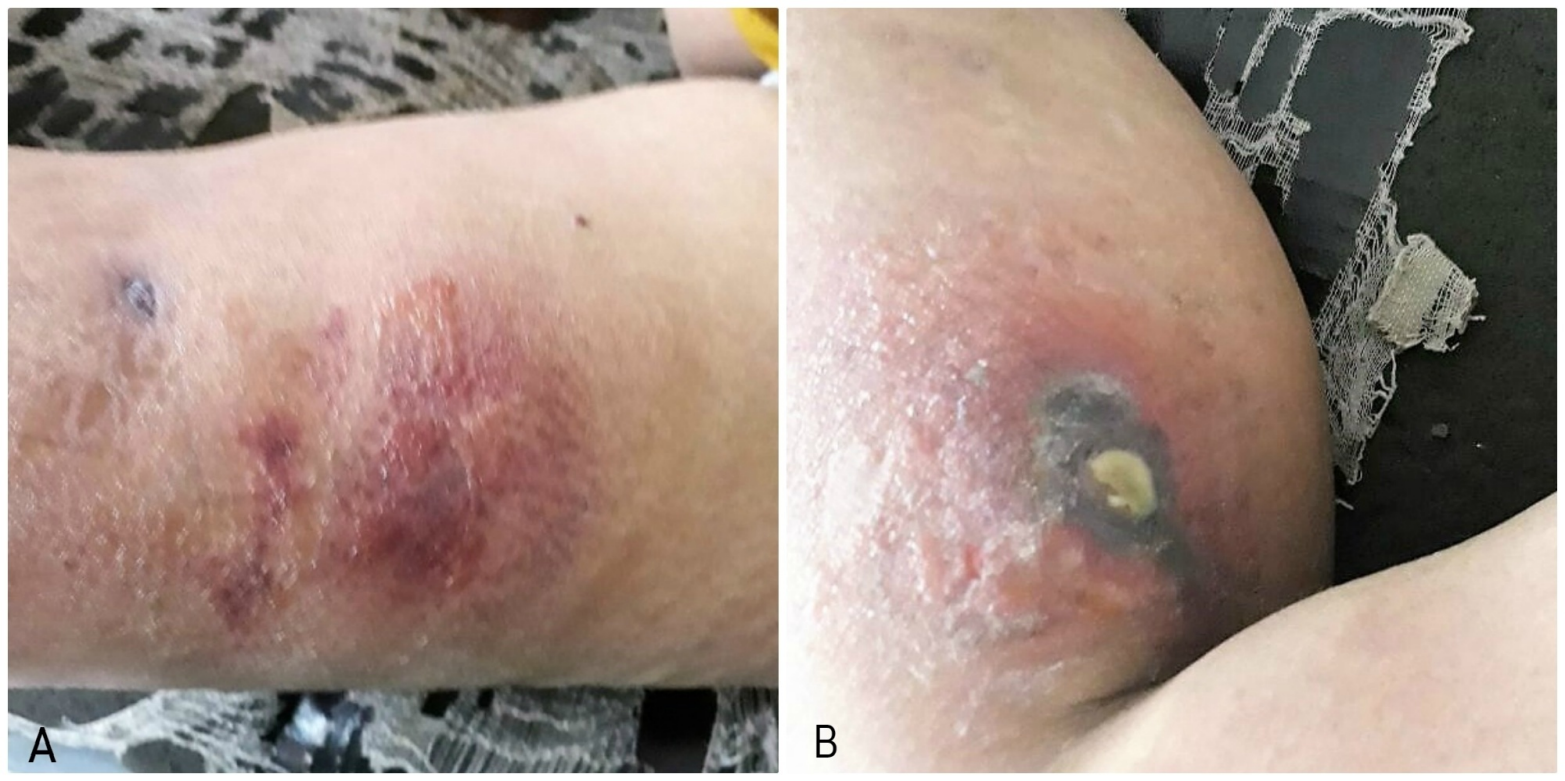
(A) shows scarred and indurated lesion on left thigh. (B) Ulcerated lesion with purulent discharge on right buttock.

The general physical and systemic examinations were unremarkable. Upon local examination of the skin, multiple ulcerated lesions with an erythematous base and edematous necrotizing centers with purulent discharge were appreciated on the left thigh, right gluteus, and left arm (Figure 1). Bilateral lower limb edema was noted. Right lower limb was hot and tender.

Complete blood count, serum biochemistry, serum electrolytes, liver function tests, renal function tests, and coagulation tests were in normal range. Urine analysis showed a pH of 6.5, leucocytes 12-16 per high power field (HPF), red blood corpuscles 20-25 per HPF, and epithelial cells 2+ per HPF. Culture and sensitivity of purulent discharge showed growth of Klebsiella and Pseudomonas aeruginosa. Chest X-ray and abdominal ultrasound were insignificant. The autoimmune assay was done (Table 1).

**Table 1:** Autoimmune assay of the patient

Subtypes of Anti-nuclear antibody	Result
Anti-Nuclear Antibodies	Negative
Serum Anti ds DNA	Equivocal (21.4)
Anti-smooth muscle antibodies	Negative
Anti-mitochondrial Antibodies	Negative
Anti-gastric parietal cell antibodies	Negative

Punch biopsy of the skin was done. The epidermis was normal. Melanophages and mild lymphoplasmacytic infiltrate was seen in the dermis. Deep dermis showed foamy histiocytes with scattered multinucleated giant cells in subcutaneous fat. Hyaline glass appearance was seen in the lobules of adipose tissue (lobular panniculitis). Based on the histological findings the diagnosis of lupus profundus (LP) was established.

We treated her with antibiotics as per sensitivity to the pus culture, oral hydroxychloroquine 400 mg daily for 12 weeks, and oral corticosteroid 30 mg daily which was tapered to 5 mg daily over six weeks when the lesions scarred with lipoatrophy. Potassium permanganate wash was applied locally on the lesions along with local methylprednisolone aceponate 5 mg daily. The patient was discharged after one week. The patient is still in follow-up for the suspected flare of lesions and also susceptibility to develop systemic lupus erythematosus. 

## Discussion

Lupus profundus is the least common subtype of CLE. It is frequent in middle-aged women [3]. The lesions are common on the face, proximal extremities, buttocks, breasts, and trunk [1, 3]. However, according to Batrani et al., there are only 15 cases in the internet-based literature with LP presenting at a non-acral site [2]. Our case is also a non-acral presentation of lupus profundus.

Clinically, LP presents with ulcerated, indurated, subcutaneous, painful or tender nodules [3]. Histological diagnosis is based on the presence of hyaline necrosis of the subcutaneous fat lobules along with lymphocytic infiltration of fat lobules. Plasma cells, lymphoid follicles, and eosinophils may also be present [4]. Presence of mucin deposition along with epidermal changes such as degeneration indicates coexistence of DLE and LP, which is seen in as many as 70% cases [5]. There were no epidermal changes in our case, hence, our diagnosis remains isolated for LP.

CLE is taken as the precursor of SLE in as many as 25% of cases. However, the risk is higher in patients with SCLE than in localized DLE [1]. Grönhagen et al. in an epidemiological study reported the incidence of CLE to be 4/100000; with a male to female ratio of 1:3. It reported 18% of CLE patients to be later diagnosed with SLE, highest diagnosis being for SCLE. On the other hand, it also reported 24% of newly diagnosed CLE patients to already have the diagnosis of SLE [6].

SLE accompanying LP is reported in as few as 2%-5% cases; 15% are able to progress to SLE and 50% have evidence of SLE. Hence, LP can be taken as an initial presenting manifestation of SLE [5]. Zhao et al. have reported only 10 cases of SLE that initially presented with LP from the internet-based medical literature search [7]. Our patient didn’t meet the SLE diagnostic criteria of American College of Rheumatology at the time of presentation [8]. However, we are following the patient in view of long-term diagnosis of SLE. Our patient had unremarkable immunological assay. Literature has reported antinuclear antibody to be positive in 27%–95% cases of LP [9-11]. Anti-double-stranded DNA antibodies are identified in fewer cases [12]. Laboratory findings also include lymphopenia, anemia, decreased C4 levels, and positive rheumatoid factor; however, in most cases, serology remains unremarkable as in our case.

The diagnosis of LP is based on clinical, serological and histopathological grounds. The differential diagnosis includes connective tissue panniculitis such as morphea profunda (MP) and inflammatory diseases of subcutaneous fat—erythema nodosum, erythema induratum. The most challenging differential diagnosis is subcutaneous panniculitis-like T-cell lymphoma (SPTCL). LP is differentiated from MP by the absence of dermal and subcutal sclerosis. SPTCL histologically presents with atypical CD3+, CD8+ and CD4-T lymphocytes with the expression of clonal a/b T cell receptors and typical localization of lymphocytes around fat cells in a rim-like manner. Plasma cells are also classically absent in SPTCL [7, 13].

We managed our patient with antimalarial therapy which is the first-line recommendation for LP [7]. For recalcitrant cases, systemic therapies such as thalidomide, dapsone, cyclosporine, and rituximab can be used [14].

## Conclusions

Ulcerated LP is a rare cause of ulcerated/indurated, painful subcutaneous plaques. LP may present as a localized entity or in association with SLE or it may lead to SLE later in life. Early diagnosis based on histopathology and aggressive treatment is essential to prevent significant physical morbidity and progression to systemic involvement. All LP cases, not diagnosed with SLE, should be followed in long-term in view of suspected progression to SLE.
